# Blood Compatibility of ZrO_2_ Particle Reinforced PEEK Coatings on Ti6Al4V Substrates

**DOI:** 10.3390/polym9110589

**Published:** 2017-11-10

**Authors:** Jian Song, Zhenhua Liao, Hongyu Shi, Dingding Xiang, Lin Xu, Yuhong Liu, Xiaohong Mu, Weiqiang Liu

**Affiliations:** 1 State Key Laboratory of Tribology, Tsinghua University, Beijing 100084, China; songj13@mails.tsinghua.edu.cn (J.S.); shihongyu1991@126.com (H.S.); xdd16@mails.tsinghua.edu.cn (D.X.); 2 Biomechanics and Biotechnology Lab, Research Institute of Tsinghua University in Shenzhen, Shenzhen 518057, China; liaozh@tsinghua-sz.org; 3 Department of Osteology, Dongzhimen Hospital Affiliated to Beijing University of Chinese Medicine, Beijing 100700, China; xulinguke@163.com

**Keywords:** blood compatibility, PEEK, zirconia (ZrO_2_), coating

## Abstract

Titanium (Ti) and its alloys are widely used in biomedical devices. As biomaterials, the blood compatibility of Ti and its alloys is important and needs to be further improved to provide better functionality. In this work, we studied the suitability of zirconia (ZrO_2_) particle reinforced poly-ether-ether-ketone (PEEK) coatings on Ti6Al4V substrates for blood-contacting implants. The wettability, surface roughness and elastic modulus of the coatings were examined. Blood compatibility tests were conducted by erythrocytes observation, hemolysis assay and clotting time of recalcified human plasma, to find out correlations between the microstructure of the ZrO_2_-filled PEEK composite coatings and their blood compatibilities. The results suggested that adding ZrO_2_ nanoparticles increased the surface roughness and improved the wettability and Derjaguin-Muller-Toporov (DMT) elastic modulus of PEEK coating. The PEEK composite matrix coated Ti6Al4V specimens did not cause any aggregation of erythrocytes, showing morphological normal shapes. The hemolysis rate (HR) values of the tested specimens were much less than 5% according to ISO 10993-4 standard. The values of plasma recalcification time (PRT) of the tested specimens varied with the increasing amount of ZrO_2_ nanoparticles. Based on the results obtained, 10 wt % ZrO_2_ particle reinforced PEEK coating has demonstrated an optimum blood compatibility, and can be considered as a candidate to improve the performance of existing PEEK based coatings on titanium substrates.

## 1. Introduction

Titanium (Ti) and its alloys have been widely used in blood-contacting medical devices, such as vascular stents, dental implants and artificial joints, owing to their excellent biocompatibility, CT/MRI compatibility and mechanical properties [1,2]. However, the main issue for these implant devices is the thrombosis introduced by biomaterials, which may lead to implant failure and revision surgery [3]. It is reported that titanium is much more thrombogenic than other conventional biomaterials such as stainless steel [4]. Meanwhile, poor tribological behavior of titanium alloys also limits its long-term service as an implant device [5]. Therefore, various biocoatings, such as hydroxyapatite (HA) [6], diamond-like carbon (DLC) [7], titanium nitrides (TiN) [8] and some polymeric coatings [9,10], have been developed to improve the tribological characteristics and hemocompatibility of Ti and Ti-based materials. Each coating has its advantages and limits. Few coatings could combine the anti-wear properties of ceramic, toughness of metal, and an optimum bonding strength and biocompatibility as a biomaterial [11].

Recently, researchers in the field of blood-contacting medical devices have shown great interest in poly-ether-ether-ketone (PEEK), which is believed to be one of the leading candidates of implant materials, because of its prominent stiffness, toughness, chemical and tribological properties [12,13]. PEEK composites have been also used as coating materials for biomedical devices [14]. In fact, PEEK composite coatings on Ti-based materials can protect substrates against further wear and decrease the release of wear debris and metallic ions to the surrounding human tissues [15]. In our previous study [16], zirconia (ZrO_2_) particle reinforced PEEK was coated on Ti6Al4V substrates to investigate their friction and wear performances. The results obtained suggested that ZrO_2_ nanoparticles filled PEEK coatings improved the tribological properties of the Ti6Al4V, and that adhesive wear and mild abrasive wear were the dominant wear and failure mechanisms for PEEK/ZrO_2_ composite coatings. Blood compatibility is one of the most important properties for biomaterials [17]. Liu et al. [18] studied the blood compatibility of carbon fibers reinforced PEEK and titanium alloy, suggesting that the two materials have no significant effects on human erythrocytes, leucocytes, and platelets. Kawasaki et al. [19] carried out the surface modification of PEEK through self-initiation graft polymerization, resulting in significant improvement in blood compatibility. However, very limited investigations were conducted about the blood compatibility of ZrO_2_-filled PEEK coatings on the Ti6Al4V substrates.

In order to further explore the potential of ZrO_2_ particle reinforced PEEK coatings for biomedical applications, five different PEEK composite coatings were deposited on Ti6Al4V substrates in the present paper. The wettability and microstructure analysis were conducted for surface characterization. The erythrocytes observation, hemolysis assay and clotting time of recalcified human plasma were carried out to explore correlations between the microstructure of the ZrO_2_-filled PEEK composite coatings and their blood compatibility.

## 2. Materials and Methods

### 2.1. Sample Preparation

Square-shaped Ti6Al4V plates (side length of 10 mm and thickness of 5 mm) were cut from larger commercial Ti6Al4V panels (TC4, Baoji Titanium Industry Co., Ltd, Baoji, Shanxi, China). The commercial powders of PEEK (VICTREX^®^ 450G, Lancashire, UK) and ZrO_2_ (Sino-Rich Material Technology Co., Ltd, Beijing, China) were purchased. The average size of PEEK powders was below 100 μm and that of ZrO_2_ was about 50 nm. 

The Ti6Al4V plates were successively ground using 60 to 1500 mesh alumina (Al_2_O_3_) abrasive papers before final polishing with diamond paste to get a mirror-like surface finish, which were used as the substrates for the deposition of the coatings. The entire deposition processes were performed by an authorized applicator according to the reported process [20]. Those polished plates were first cleaned in an acetone bath for 15 min and degreased in a water-based solution of alkaline cleaner for 10 min followed by thorough rinsing with deionized water, respectively [21]. Then, the cleaned substrates were grid-blasted using an 80-grit aluminum oxide abrasive to increase the surface roughness and make the mechanical bonding of the deposited coatings better. Morpholine and xanthan gum were mixed with these powder compounds into a liquid dispersion to avoid rusting rust and resist sagging (formation of tears of the deposited coating). Five different polymeric coatings, namely, PEEK containing 0, 2, 5, 10 and 15 wt % ZrO_2_ nanoparticles were deposited on the grit-blasted substrates using a spray gun. Next, the specimens were cured above 350 °C to remove the gum particles from the coatings. As a result, only the solid particles of PEEK and ZrO_2_ were finally left in the coating. In this study, the PEEK based coatings were used without polishing the surface. As shown in Table 1, the specimens coated with PEEK containing 0, 2, 5, 10 and 15 wt % ZrO_2_ nanoparticles were defined as S1, S2, S3, S4 and S5, respectively. 

### 2.2. Surface Characterization

In order to understand the thickness values of the coatings, the specimens were inlaid by epoxy resin and the cross sections were ground using 60 to 1500 mesh alumina (Al_2_O_3_) abrasive papers before final polishing with diamond paste to get a mirror-like surface finish [22]. The height images and Derjaguin-Muller-Toporov (DMT) modulus (Young’s modulus according to the DMT mode) maps of the specimens were observed by atomic force microscope (AFM, Dimension Icon, Bruker, CA, USA) with TAP525A probes (Bruker) in PEAKFORCE Quantitative Nanomechanical Mapping (Peakforce QNM) mode at ambient conditions (Relative Humidity = 17 ± 2%, Temperature = 26 ± 1 °C) [23]. The radius curvature and spring constant of the tips used in the experiment were calibrated according to the calibration procedures given in the Peakforce QNM user guide. The scan area of topology and elastic modulus maps for each sample were 5 × 5 μm^2^. The applied normal load used in Peak Force QNM was 50 nN. The scanning resolution was 512 × 512 pixels, and the scanning frequency was 1 Hz. The Poisson’s ratio of PEEK coatings was used as 0.388 for the DMT modulus [24]. The DMT modulus were calculated from the histograms of peak values in the DMT modulus maps by the software “Nanoscope Analysis” (v 1.8, Bruker), respectively. The surface roughness values (Ra) were evaluated using a 3D white-light interfering profilometer (MicroXAM 3D, ADE Corp). In order to investigate the wettability of the PEEK/ZrO_2_ coatings, the static contact angle values were measured using a contact angle goniometer (Data Physics Corporation, San Jose, CA, USA) at ambient temperature. One drop (3 μL) of distilled water was dropped on the surface of the tested samples with an automatic piston syringe and the photograph was captured. All experimental results were obtained by averaging the values of at least four repetitions.

### 2.3. Blood Donors

The quality of the blood is of critical importance for the blood compatibility test. Thus, the following exclusion criteria for the blood donors were strictly applied in this study: smokers, drug-taking in the last 2 weeks, especially oral contraceptives, acetylsalicylic acid, non-steroidal antiphlogistics and others hemostasis-affecting agents [25]. Blood for this study was obtained from healthy volunteers with an activated partial thromboplastin time (APTT) in a normal range (age: >20 and <40 years), which was mixed with acid citrate dextrose.

### 2.4. Erythrocytes Observation

Fresh anticoagulated blood (2 mL) was incubated with those PEEK based coatings at 37 °C for 20 min, respectively [26]. The one without any samples was used as blank control. After incubation, the shape and aggregation of erythrocytes were examined under an inverted microscope (Motic AE30, Motic Electric Group Co., Ltd., Xiamen, China) at high magnification (400×). One representative image for each specimen was given.

### 2.5. Hemolysis Assay

Fresh anticoagulated blood from human volunteers (2 mL) was diluted with 2.5 mL of normal saline [27]. The 0.2 mL diluted blood and 10 mL normal saline were mixed with tested samples. The positive controls consisted 0.2 mL blood with 10 mL distilled water and the negative controls consisted 0.2 mL blood with 10 mL normal saline. The mixture was kept at 37 °C for 60 min and then centrifuged at 2500 rpm for 5 min [28]. The supernatant was transferred to a 96-well plate. The absorbance was evaluated using a BioTek synergy 2 Multi-Mode Microplate Reader at 545 nm. The hemolysis rate (HR) was calculated as shown in Equation (1):

(1) $$HR=\frac{OD_{t}-OD_{nc}}{OD_{pc}-OD_{nc}}\times 100\%$$

where *OD_t_*, *OD_pc_* and *OD_nc_* were the absorbency of test specimens, positive control and negative control, respectively. All data were obtained based on the average of four repeats.

### 2.6. Plasma Recalcification Time

The thrombus formation was evaluated according to the measurement of plasma recalcification time (PRT) of the ZrO_2_-filled PEEK coatings. Before the PRT experiments, platelet-poor plasma (PPP) was prepared by centrifuging the whole blood at 3000 rpm for 15 min [29]. The PPP and 25 mM CaCl_2_ aqueous solution were placed in water bath at 37 °C for 30 min. The tested specimens were added into test tubes, respectively. The 1 mL preheated PPP was dropped on each tested specimen in a 24-well plate and incubated statically at 37 °C for 1 min. Then, the preheated 1 mL CaCl_2_ aqueous solution (25 mM) was added into the cell of the 24-well plate and the stopwatch was started simultaneously. A stainless steel wire hook was dipped into the plasma to monitor clot formation. PRT was recorded at the first sign of a fibrin blood clot appeared [30]. The blank control was consisted 1 mL PPP and 1 mL 25 mM CaCl_2_ aqueous solution. The test was repeated four times for each sample to get a reliable value.

### 2.7. Statistics

Following the above analyses, One-way ANOVA and Least-Significant Difference (LSD) post hoc tests were conducted in “SPSS” (SPSS^®^ v. 18, SPSS Inc., Chicago, IL, USA) to compare the contact angles, HRs and PRTs of the tested samples. A level of *p* < 0.05 was considered statistically significant.

## 3. Results

### 3.1. Surface Characterization

**Figure 1:**
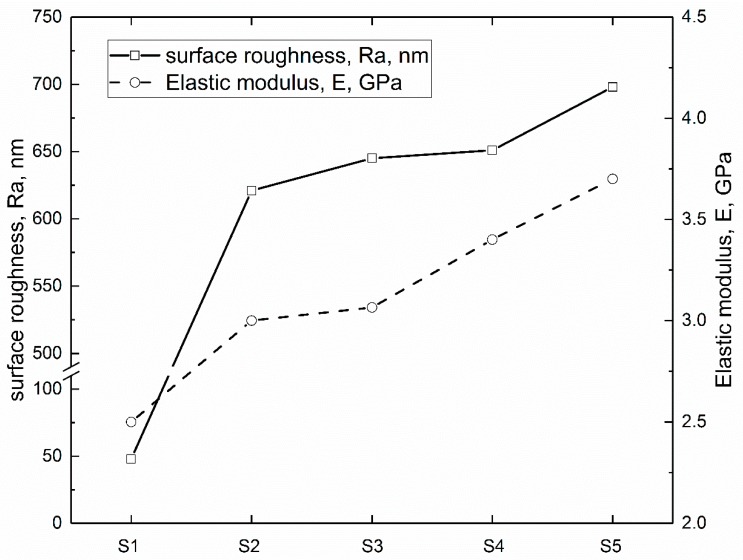
Variations of surface roughness (Ra) and Derjaguin-Muller-Toporov (DMT) elastic modulus (E) of the PEEK based coatings.

It is reported that surface roughness is a key factor in increasing the blood compatibility of the implants [31]. In this study, the surface roughness values (Ra) were measured by a 3D white-light interfering profilometer and the elastic modulus of the deposited coatings were determined by AFM. The variations of the surface roughness and elastic modulus of the PEEK based coatings are displayed in Figure 1, which can be regarded as the composites as a function of the ZrO_2_ particle content in wt %. According to our previous characterization [16], the introduced ZrO_2_ nanoparticles are distributed evenly in PEEK matrix and the thickness values of the PEEK based coatings were around 25 μm, suggesting that the measured surface roughness and elastic modulus should not be affected by the substrates. It is indicated that the surface roughness values of PEEK composite coatings increase with the increment of ZrO_2_ particle content. The surface roughness values are 48, 621, 645, 651 and 698 nm for S1 to S5 samples, respectively. Regarding the DMT modulus of the PEEK based composite coatings, the continuous increasing trend of the elastic modulus up to 15 wt % ZrO_2_ nanoparticles as displayed in Figure 1. The highest increment occurs in S5, which raising the PEEK modulus from 2.5 up to 3.7 GPa (an increment percentage of 48%). It is reported that the elastic modulus of composite polymeric matrix is a function of properties of constituents, volume fraction of components [32]. Thus, the greater adhesion between the PEEK and ZrO_2_ nanoparticles causes less debonding when a stress is applied, leading to the improvement of elastic modulus. The increase in modulus is also attribute to the modulus of ZrO_2_, E = 220 GPa [33], which is much greater than that of the PEEK matrix. Therefore, one may conclude that the blended ZrO_2_ nanoparticles could increase the DMT elastic modulus. Owing to the different calculate modes, the elastic modulus of PEEK varies from 2.6 to 4.0 GPa in previous literatures [34,35], indicating the results obtained in this study are in a reasonable range.

**Figure 2:**
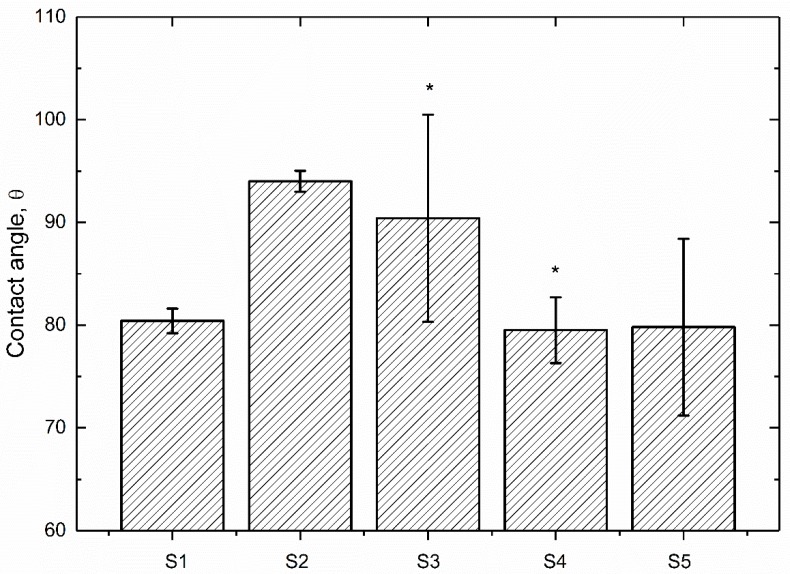
Contact angles of PEEK based coatings under distilled water (N = 4, * *p* < 0.05, statistically significant difference; S1 vs. S2 to S5).

It has been reported that the surface wettability is a key factor of the biological response for biomaterials [36]. The wettability of PEEK based coating were evaluated by measuring the contact angle under distilled water and the results obtained are set out in Figure 2. As can be seen, the contact angle of pure PEEK coating under distilled water is 80.4°, which is similar to the results found in previous literature [37]. The contact angles of PEEK/ZrO_2_ coatings decrease with the increment of ZrO_2_ nanoparticles content, indicating that those nanoparticles have improved the wettability of PEEK coating. The statistics analysis indicates that there is a significant difference (*p* < 0.05) between the contact angle values of S1 and S3/S4 samples, respectively. In particular, the contact angle of S5 is the lowest (79.8°), which demonstrates a decline of 15.1% in comparison with that of S2. It is suggested that the contact angle value of a material means the wettability for a surface, which could be affected by surface topography and chemistry [37]. Figure 2 also indicates larger variations in contact angle values of the ZrO_2_ particle reinforced PEEK coatings in comparison with the pure one. Those ZrO_2_ nanoparticles increased the surface roughness (Figure 1) and the oxide in ZrO_2_ enhanced water adhesion tension and as a result, the water contact angles of ZrO_2_-filled PEEK coatings were declined with the increasing ZrO_2_ nanoparticles content.

### 3.2. Erythrocytes Observation

**Figure 3:**
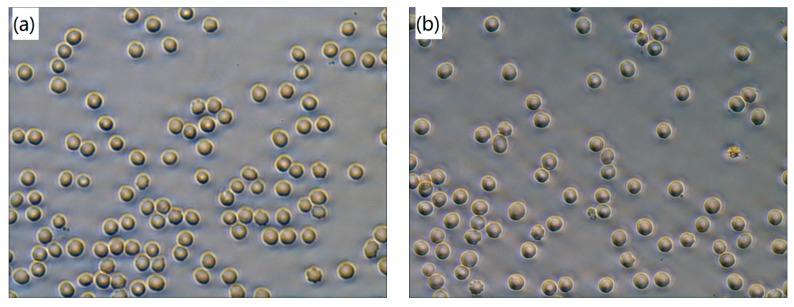

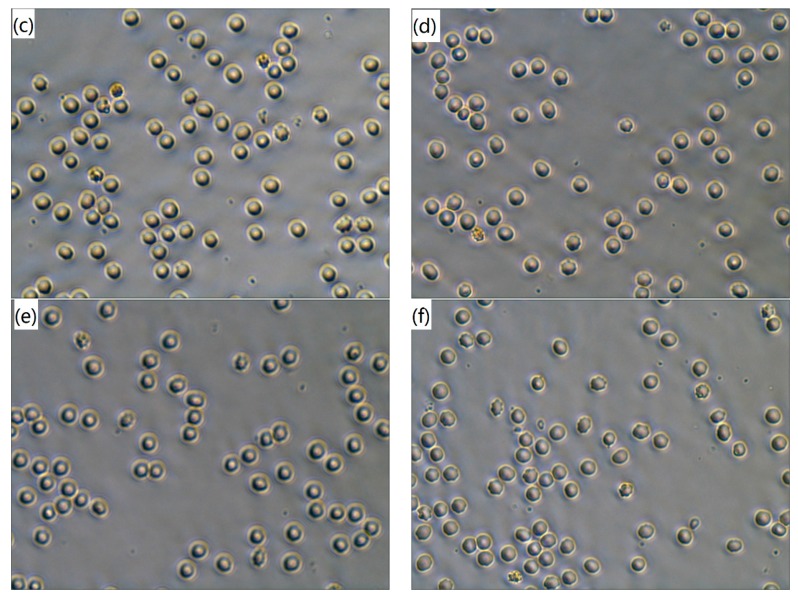
Optical micrographs of human blood red cells in whole blood after 20 min incubation with different specimens: (**a**) blank control; (**b**) S1; (**c**) S2; (**d**) S3; (**e**) S4 and (**f**) S5. All images are at 400× magnification.

Erythrocyte interaction with polymers plays an important role in the biosafety of implant biomaterials [26]. In order to analyze the effect on erythrocytes shape and aggregation, the erythrocytes in whole blood incubated with PEEK/ZrO_2_ coatings were observed microscopically in this study. As demonstrated in Figure 3, there is no aggregation of erythrocytes in whole blood after 20 min incubation with all tested specimens, which is also morphological normal and in accordance with the blank control sample (Figure 3a). Hence, it can be concluded that the PEEK composite matrix coated Ti6Al4V specimens cannot cause any aggregation and shape change of the erythrocytes, which is similar to the control sample, indicating good blood compatibility. The same results are also visible with the hemolytic properties of those tested specimens.

### 3.3. Hemolysis Assay

**Figure 4:**
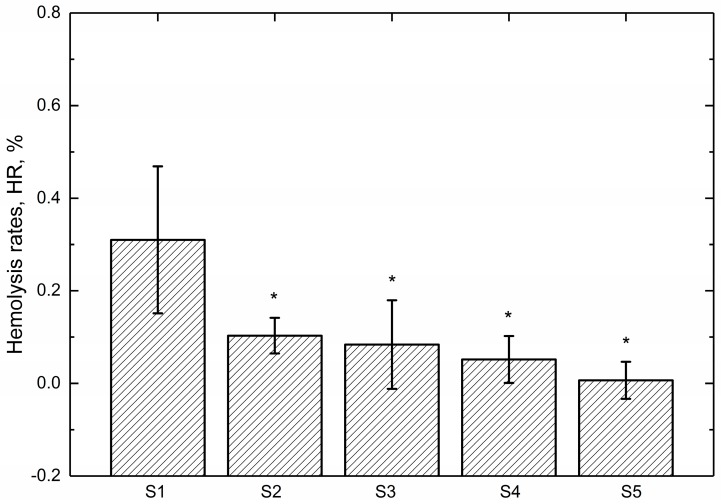
Hemolysis rates of the tested specimens (N = 4, * *p* < 0.05, statistically significant difference; S1 vs. S2 to S5).

Hemolysis assay is widely used as a useful and reliable way to evaluate the blood compatibility of biomaterials [38]. The hemolysis rate (HR) values of the tested specimens are shown in Figure 4. The smaller the HR value, the better the blood compatibility of a biomaterial is. It can be seen that the HR values of all the samples are much less than 5%, the safe value for biomaterials according to ISO 10993-4 standard, indicating that the tested samples are nonhemolytic when contacted with blood [39]. Figure 4 also reveals that the HR values of the tested specimens decrease with the increasing amount of ZrO_2_ nanoparticles. Meanwhile. the statistics analysis of the obtained HR values indicates that there is a significant difference (*p* < 0.05) between pure PEEK coating (S1) and ZrO_2_-filled PEEK coatings (S2 to S5), suggesting that introduced ZrO_2_ nanoparticles affected the hemolysis properties and then improves the blood compatibility of PEEK coating. One possible explanation for it might be that the surface roughness of these coated samples have been increased with the increasing amount of ZrO_2_ nanoparticles, leading to the reduction of blood cells adhesion. As a result, the injury of blood cells caused by the coatings decreased, leading to decline of the HRs of PEEK composite coatings. Furthermore, the results obtained are in agreement with the optical micrographs observed by inverted microscope displayed in Figure 3.

### 3.4. Plasma Recalcification Time

**Figure 5:**
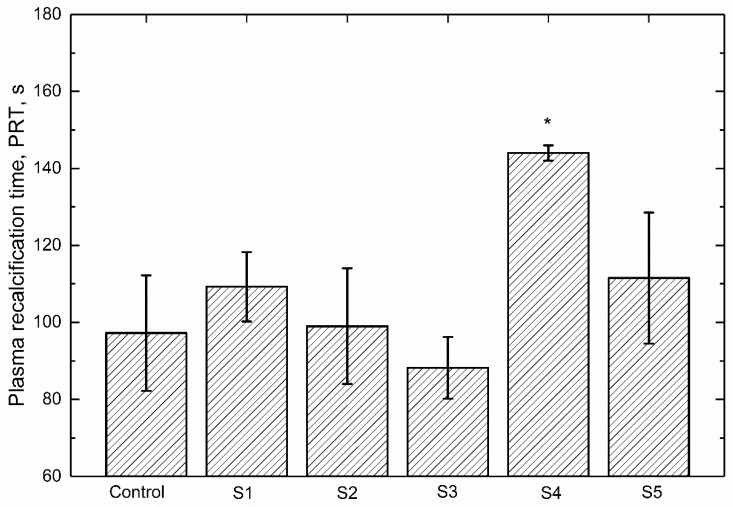
Plasma recalcification time of different samples (N = 4, * *p* < 0.05, statistically significant difference; Control vs. S1 to S5).

It is reported that the plasma coagulation characteristics of a biomaterial represent its potential to react with blood component and possibility to cause thrombosis [40,41]. Plasma recalcification time (PRT) is a way to measure the time taken for clotting of blood or to determine the deficiency of factor responsible for clotting. It is an evaluation of the intrinsic coagulation cascade activation defined by the time for fibrin clot formation when calcium has been reintroduced into sodium citrate anticoagulated plasma [42]. Hence, the PRT assay was conducted to further evaluate the blood compatibility of ZrO_2_-filled PEEK composite coatings in this study. The PRTs of different samples are illustrated in Figure 5. The one without specimens is defined as blank control. It can be seen that the blank control obtains a PRT of 97.2 s. It is indicated that PRTs of the tested specimens vary with the increasing number of ZrO_2_ nanoparticles. In detail, the S3 and S4 samples demonstrate the shortest (88.2 s) and the longest (144.0 s) PRT value, respectively. It has been suggested that longer PRT often indicates better blood compatibility of the material in contact with blood [43]. As displayed in Figure 5, the statistics analysis reveals that there is a significant difference between the blank control and S4 sample. S4 has a significant increase in PRT, which is about 142.01% of that for pure PEEK coating (S1), suggesting that the reinforced 10 wt % ZrO_2_ nanoparticles play an important role in restricting the transformation of fibrinogen and could decrease the activation of intrinsic coagulation system [44]. Therefore, it can be concluded that the blood compatibility of PEEK coating can be enhanced effectively by adding 10 wt % ZrO_2_ nanoparticles. Furthermore, the PRTs observed in the present study matched well with the ones reported in previous literature [43,45].

## 4. Discussion

The implant biomaterials must possess suitable wettability and good blood compatibility [46]. The wettability of PEEK based coatings is assessed through contact angle. The blood compatibility of those coatings is evaluated through a series of testing. The destructive effect of the tested specimens to the erythrocyte were observed microscopically. The hemolysis assay was carried out to confirm the observation of erythrocyte. Furthermore, the anticoagulant property was evaluated through PRT. Generally, HR has to be less than 5% (ISO 10993-4) and long PRT is always desired.

The obtained results of surface characterizations shown in Figure 1 and Figure 2 indicate that the addition of ZrO_2_ nanoparticles into PEEK matrix contributes to the increment in the surface roughness, reduction in contact angle and the improvement of Young’s modulus in comparison with pure PEEK coating (S1), decreasing cell adhesion and proliferation, which are benefit to the blood compatibility of biomaterials [44].

Regarding the hemolysis tests, Figure 3 indicates that no aggregation and shape change of the erythrocytes can be found, which indicates that the PEEK based coatings causes no damage to erythrocyte. Figure 4 clearly proves that the HRs of the tested samples are lower than the recommended value (5%), which decrease significantly in comparison with the reported value of bare Ti6Al4V (1.5%) [47]. The increase of surface roughness and decrease of contact angle might be ascribed for the further decrease in the HRs of ZrO_2_-filled PEEK coatings, which are in agreement with previous literature [48,49]. 

The PRT, an indicator of the intrinsic coagulation cascade activation, was measured to further study the blood compatibility of those PEEK based coatings. It is reported that the ZrO_2_ particles showed less toxicity and higher antioxidant activity in nano form than their micron counterpart [50], suggesting the excellent biocompatibility of ZrO_2_ as a kind of introduced particles in polymeric matrix. As can be found in Figure 5, the PRT of S4 is the highest (144.0 s), which is significantly different with that of blank control, suggesting that the blood-clotting factors are more difficultly activated on the surface of 10 wt % ZrO_2_-filled PEEK coating [51]. Yahapour et al. [52] found a reduction in thrombin generation on titanium and glass surfaces with hydrophilic modifications compared to hydrophobic coatings. Owing to the improved wettability and increasing surface roughness, the thrombin generation and cell adhesion of S4 and S5 are decreased, prolonging the PRTs in comparison with that of blank control.

Based on the results presented above, it is clear that the blood compatibility of PEEK based coatings are prominent. However, for the real time application of these coatings on the biomedical Ti6Al4V material, the long-term in vivo tests on the biocompatibility using rats and/or mice need to be performed and analyzed.

## 5. Conclusions

The blood compatibility of these ZrO_2_ particle reinforced PEEK coatings was evaluated using erythrocytes observation, hemolysis assays and thrombus formation analysis. According to the results of this study, the addition of ZrO_2_ nanoparticles increased the surface roughness and improved the wettability and DMT elastic modulus of PEEK coating_._ Under comprehensive investigations in the present study, 10 wt % ZrO_2_ particle reinforced PEEK coating has demonstrated an optimum blood compatibility, which has a great potential in the medical application of PEEK composite coatings on titanium alloy.

## Figures and Tables

**Table 1:** The poly-ether-ether-ketone (PEEK) based composite coatings on Ti6Al4V substrate.

Sample Code	Coating
S1	Pure PEEK
S2	PEEK + 2 wt % ZrO_2_
S3	PEEK + 5 wt % ZrO_2_
S4	PEEK + 10 wt % ZrO_2_
S5	PEEK + 15 wt % ZrO_2_
